# Metabolic Portraits of Breast Cancer by HR MAS MR Spectroscopy of Intact Tissue Samples

**DOI:** 10.3390/metabo7020018

**Published:** 2017-05-16

**Authors:** Tonje H. Haukaas, Leslie R. Euceda, Guro F. Giskeødegård, Tone F. Bathen

**Affiliations:** Department of Circulation and Medical Imaging, Faculty of Medicine and Health Sciences, Norwegian University of Science and Technology (NTNU), 7491 Trondheim, Norway; tonje.h.haukaas@ntnu.no (T.H.H.); leslie.e.wood@ntnu.no (L.R.E.); guro.giskeodegard@ntnu.no (G.F.G.)

**Keywords:** amino acid metabolism, breast cancer, choline phospholipids, glycolysis, HR MAS MR spectroscopy

## Abstract

Despite progress in early detection and therapeutic strategies, breast cancer remains the second leading cause of cancer-related death among women globally. Due to the heterogeneity and complexity of tumor biology, breast cancer patients with similar diagnosis might have different prognosis and response to treatment. Thus, deeper understanding of individual tumor properties is necessary. Cancer cells must be able to convert nutrients to biomass while maintaining energy production, which requires reprogramming of central metabolic processes in the cells. This phenomenon is increasingly recognized as a potential target for treatment, but also as a source for biomarkers that can be used for prognosis, risk stratification and therapy monitoring. Magnetic resonance (MR) metabolomics is a widely used approach in translational research, aiming to identify clinically relevant metabolic biomarkers or generate novel understanding of the molecular biology in tumors. Ex vivo proton high-resolution magic angle spinning (HR MAS) MR spectroscopy is widely used to study central metabolic processes in a non-destructive manner. Here we review the current status for HR MAS MR spectroscopy findings in breast cancer in relation to glucose, amino acid and choline metabolism.

## 1. Introduction

Breast cancer is by far the most common cancer in women worldwide [1]. In Norway, there were 3415 diagnosed breast cancer cases among women in 2015 [2], making it the most frequent neoplasm in this population group. Although the estimated five year survival in breast cancer patients in Norway is almost 90% [2], predicting patient outcome is still a major challenge. Patients with similar clinical diagnosis might have different prognosis and response to treatment due to the heterogeneity and complexity of the disease.

**Figure 1:**
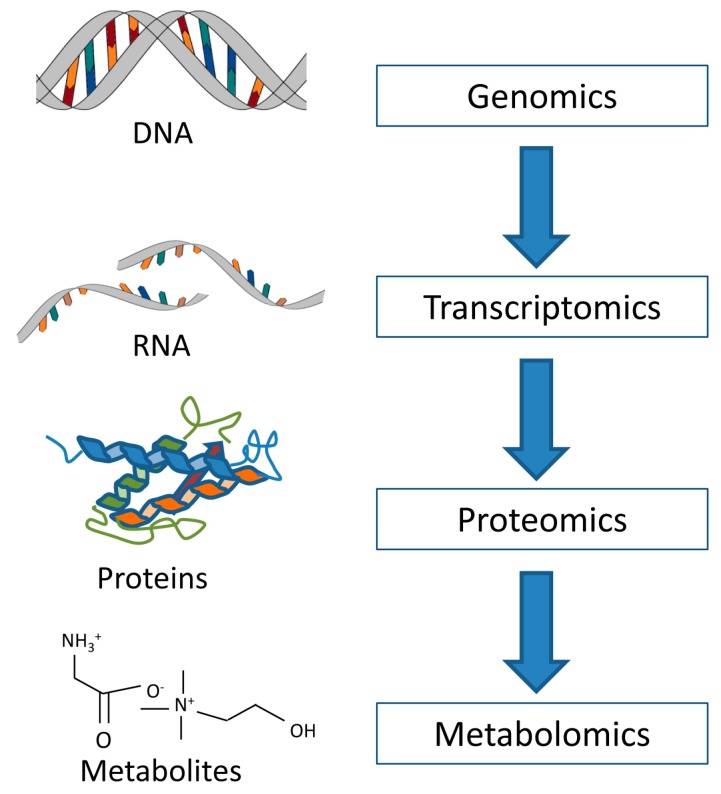
The omics cascade. Metabolomics is the final step in the cascade, and thus studies information closer to the phenotype than the preceding omics.

Breast cancer heterogeneity is manifested at different molecular, i.e., omics levels (Figure 1). Transcriptomics studies gene expression by measuring mRNA, the transcripts of DNA. At the mRNA level, five intrinsic subtypes of breast cancer have been identified: luminal A, luminal B, basal-like, HER2-enriched and normal-like breast cancer [3,4]. These subtypes have been found to correlate with survival, with the luminal A subtype having the best prognosis while the basal-like subtype has the worst prognosis and is considered the most aggressive [4].

At the proteomic level, differences in the expression of estrogen and progesterone receptors (ER and PgR, respectively), human epidermal growth factor receptor 2 (HER2) and the nuclear protein Ki-67 play an important role in current breast cancer clinical decision-making in terms of prognosis and optimal treatment plan. ER and PgR are transcription factors that activate important proliferation processes and production of growth factors upon binding with their ligands (the hormones estrogen and progesterone, respectively) [5]. Since ER activation also regulates the PgR-gene, less than 1% of PgR-positive (PgR+) cases are ER-negative (ER-) [6]. Over-expression of ER and/or PgR are found in approximately 70–80% of all breast cancer cases [7,8]. Due to the tumor’s dependency on hormonal stimuli, these patients are likely to benefit from endocrine therapy, and have a better prognosis than hormone receptor negative patients [7]. Over-expression of the tyrosine kinase associated receptor HER2, and amplification of its gene ERBB2, is found in 15–23% of all breast cancers [9]. HER2 over-expression is associated with aggressiveness and poorer prognosis. However, targeted anti-HER2 treatment improves progression-free survival and overall survival for these patients [10]. Ki-67 is present in proliferating cells, and is considered a marker of proliferation. Its level is also associated with breast cancer prognosis and may be included in clinical decision-making [11].

Breast tumors not expressing ER, PgR and HER2 are classified as triple negative breast cancer (TNBC). This breast cancer subtype is biologically aggressive, is associated with a poor prognosis and is considered a clinically, histologically and molecularly heterogeneous disease [12,13]. For TNBC patients, chemotherapy is often a mandatory inclusion in the treatment plan, since these tumors are unresponsive to endocrine and anti-HER2 therapy. Furthermore, six breast cancer subtypes based on protein expression by reverse-phase protein array (RPPA) have been identified [14,15]. These protein subtypes display considerable overlap with the gene-expression intrinsic luminal A, luminal B, HER2-enriched and basal-like subtypes. In addition, two novel protein-defined subtypes were identified and termed reactive due to many of their characteristic proteins probably being produced by the tumor microenvironment. The reactive I protein subtype appeared as a subset of the luminal A intrinsic subtype while the reactive II protein subtype is a combination of intrinsic subtypes.

Although research has contributed to the discovery of several subgroups of breast cancer as stated above, there are still numerous unresolved aspects of breast cancer heterogeneity leading to inefficient treatment of patients [16]. Better understanding of the heterogeneous biology of breast cancer could contribute to finding new personalized treatments. One area that may contribute to better understanding of breast cancer is metabolomics. Reprogramming of energy metabolism is necessary for tumor survival, growth and proliferation [17]. This field has gained increasing interest as it has been found that signaling pathways controlled by genes of particular relevance to cancer (oncogenes, tumor suppressors and DNA repair genes) orchestrate this metabolic switch [17,18,19]. The purpose of this deregulation is to provide cancer cells with three basic needs: (1) rapid generation of ATP as a source of energy; (2) increased synthesis of the four types of macromolecules: lipids, carbohydrates, proteins and nucleic acids and (3) proper redox stability [17].

As metabolites are downstream of genes, RNA and proteins, metabolite levels can be considered the ultimate response to genetic modifications or environmental changes including disease, drug and dietary influences. Metabolomics therefore studies information closer to the observable biological endpoints, i.e., the phenotype, when compared to the other omics levels. Depending on ongoing cellular processes and on external factors, the metabolic profile will change dynamically. By examining the metabolic portraits of breast cancer tissue, one can get a better understanding of complex biological interactions or find new biomarkers related to specific subgroups of this disease [20]. The two main analytical techniques within metabolomics are magnetic resonance (MR) spectroscopy and mass spectrometry (MS), which are complementary techniques. This review focuses on relevant findings on metabolic profiling of intact breast cancer tissue using high resolution magic angle spinning (HR MAS) MR spectroscopy, supported by in vitro MR and MS findings.

MR spectroscopy of solids and semi-solid material (e.g., tissue), is characterized by anisotropic interactions between nuclei, that arise due to the reduced mobility of molecules within these samples. This gives rise to broad peaks, possibly concealing relevant spectral information. The same does not occur in liquid solution state, as the rapid movement of the molecules averages out the anisotropic interactions. Rapid spinning of a solid sample on its axis at an angle of 54.7° with respect to the external magnetic field, referred to as the magic angle, mimics a liquid solution state in which the anisotropy of the interactions is averaged to zero [21]. This method is called HR MAS MR spectroscopy, and produces spectra of comparable resolution to that of conventional liquid solution MR spectroscopy.

Ex vivo HR MAS MR spectroscopy is widely used to study central metabolic processes related to cancer progression. It gives qualitative and quantitative metabolic information from biological tissue with minimal sample preparation. MS analysis, on the other hand, requires sample pretreatment, e.g., derivatization and extraction, which can affect the metabolic profile. The metabolites detected in intact, fresh frozen tissue using HR MAS MR spectroscopy can be considered less likely to be a product of changes other than the innate biological processes of the tumor. A study showed that a delay of freezing breast tumor tissue samples of up to 30 min after resection did not significantly change the levels of individual metabolites measured by HR MAS MR spectroscopy [22]. In addition, HR MAS MR spectroscopy is a non-destructive technique allowing subsequent analysis, for example histopathological examination or gene expression profiling, of the tissue after MR spectroscopy. Different analytical platforms have seen advances that facilitate the high-throughput collection of large amounts of data from different molecular levels. The availability of multi-layer omics data provides a unique opportunity to uncover a more comprehensive picture of biological systems. As the different molecular levels are complementary, integrating omics data, rather than focusing on data from a single omics layer, has the potential to improve models for clinical applications. Metabolic profiling alone and in combination with complementary methods thus provides important information on cancer biology, making HR MAS MR spectroscopy an attractive method due to its non-destructive nature [23].

One of the challenges when analyzing intact breast tissue using HR MAS MR spectroscopy is that tumor biopsies obtained from breast cancer patients might contain fractions of adipose tissue. Signals arising from adipose tissue cannot be separated from tumor cell lipids. Furthermore, the aliphatic side chains of fatty acids within this tissue can give rise to large and broad peaks in MR spectra, potentially overlapping with and influencing signals from important small metabolites. This effect can be limited by using an appropriate MR pulse sequence, e.g., the Carr-Purcell-Meiboom-Gill (CPMG) sequence, which filters out signals from larger molecules such as lipids. However, absolute quantification using CPMG spectra can be misleading unless proper action is taken, as metabolite peak intensities can differ slightly due to variations in T2 relaxation times [24]. In order to ensure accurate absolute quantification, the T2 relaxation effect should be corrected for [25].

**Figure 2:**
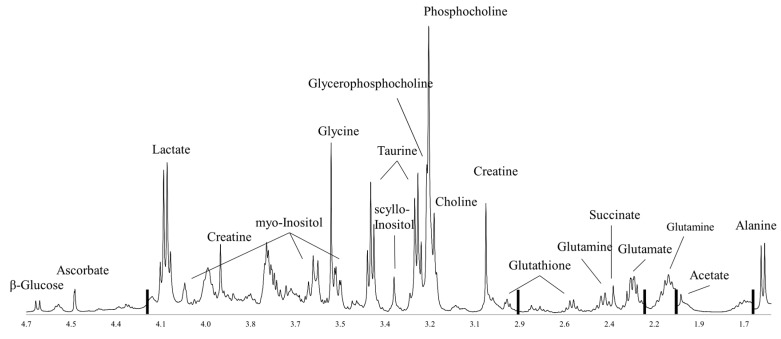
A representative proton HR MAS MR breast tumor tissue spectrum acquired using a Carr-Purcell-Meiboom-Gill (CPMG) pulse sequence on a 600 MHz spectrometer. Black bars represent excluded lipid regions. The spectral region shown excludes the water signal and above (>4.7 ppm) and regions with characteristically high and broad lipid signals (<1.4 ppm).

An important limitation of MR spectroscopy is its relatively low sensitivity (micromolar range compared to picomolar range for some MS-based methods), and thus the relatively few detectable metabolites. Using proton MR spectroscopy in vivo, only a total choline (tCho) peak can be detected. Yoon et al. however, showed that metabolites measured ex vivo using HR MAS MR spectroscopy are correlated with in vivo imaging parameters obtained using MR imaging and positron emission tomography (PET)-computerized tomography (CT) [26]. Analyzing breast cancer tissue, proton HR MAS MR spectroscopy has identified more than 30 metabolites [27]. Many of these are involved in pathways known to be important in cancer development and progression, predominantly glucose metabolism, amino acid metabolism and choline phospholipid metabolism, which are discussed in this review. A representative proton HR MAS MR spectrum from a breast cancer biopsy is illustrated in Figure 2.

## 2. Glucose Metabolism

Glucose is the major source for energy and carbon within mammalian cells and has three major metabolic fates: glycogen synthesis, glycolysis or the pentose phosphate pathway (PPP) [28]. Glycogen is the storage form of glucose in cells and has been found to be accumulated in cancerous cells [29] and in the tumor microenvironment, suggesting aberrant glycogen metabolism as a possible therapeutic target [30,31].

**Figure 3:**
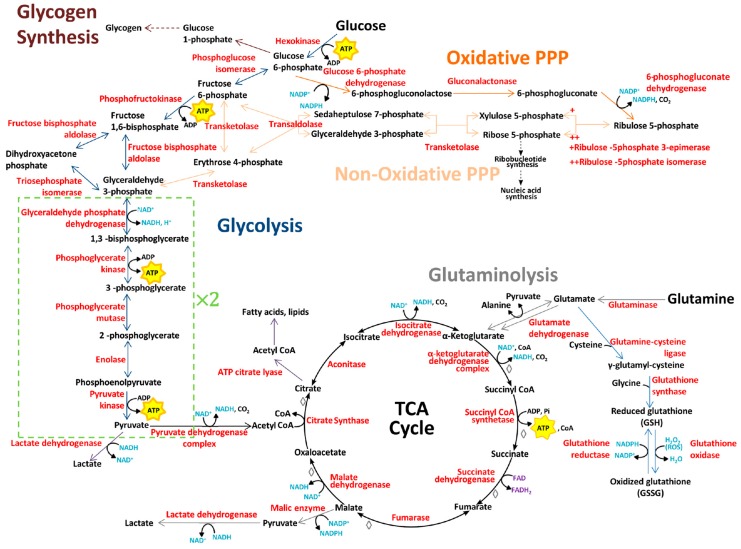
Overview of cellular glucose and glutamine metabolism. Glucose can undergo three fates: glycolysis (dark blue), glycogen synthesis (brown), or the pentose phosphate pathway (PPP), which has an oxidative (dark orange) and non-oxidative (light orange) phase. The TCA cycle (black) reactions that are recruited by glutaminolysis (gray) are marked ◊. Enzymes are shown in red. ADP: adenosine diphosphate; ATP: adenosine triphosphate; CoA: coenzyme A; FAD: oxidized flavin adenine dinucleotide; FADH_2_: reduced flavin adenine dinucleotide; NAD^+^: oxidized nicotinamide adenine dinucleotide; NADH: reduced nicotinamide adenine dinucleotide; NADP^+^: oxidized nicotinamide adenine dinucleotide phosphate; NADPH: reduced nicotinamide adenine dinucleotide phosphate; P_i_: inorganic phosphate; ROS: reactive oxygen species.

Glycolysis, or the breakdown of glucose, results in the end product pyruvate in addition to ATP and the reduced form of nicotinamide adenine dinucleotide (NADH) [19]. Pyruvate can follow one of two pathways depending on the presence or absence of oxygen. Under aerobic conditions, pyruvate enters the mitochondria where it is converted to Acetyl-coenzyme A (Acetyl CoA) by the pyruvate dehydrogenase complex (PDC). The Acetyl CoA then enters the tricarboxylic acid (TCA) cycle which yields CO_2_ as a byproduct, as well as NADH and the reduced form of flavin adenine dinucleotide (FADH_2_). The NADH and FADH_2_ produced during glycolysis and the TCA cycle are high-energy electron carriers that enter oxidative phosphorylation, also known as the electron transport chain. During oxidation of NADH and FADH_2_, electrons are transferred from these coenzymes to oxygen and free energy is released. Glycolysis, the TCA cycle and oxidative phosphorylation yield two, two and thirty-two molecules of ATP, respectively (Figure 3), and together comprise cellular respiration.

When little or no oxygen is available to oxidize pyruvate and NADH produced during glycolysis, pyruvate is quickly reduced to lactate via the action of lactate dehydrogenase (LDH). The oxidized form of NAD produced through this reaction fuels glycolysis creating a positive feedback loop (Figure 3). Although the production of ATP via anaerobic glycolysis is 100 times faster than oxidative phosphorylation [32], it is much less efficient as it yields only two molecules of ATP from glycolysis per glucose molecule. In both cancer cells and normal rapidly-proliferating cells, most of the pyruvate produced during glycolysis is converted to lactate whether oxygen is present or not; hence, their metabolism is termed aerobic glycolysis [19]. This metabolic switch is known as the Warburg effect, and was first observed by Otto Warburg [33]. The reduced efficacy to generate ATP has been suggested to be an adaption to facilitate the uptake and incorporation of nutrients into biomass needed to produce a new cell [19]. It is also suggested that the production of lactate is favorable to tumor cells, making them more destructive for the surrounding tissue and more resistant against the immune system [34]. To compensate for the inefficient ATP production, most tumors have an increased rate of glucose uptake. This property makes PET a highly sensitive and specific clinical tool to identify primary and metastatic lesions [35,36]. This technique uses the glucose analogue tracer ^18^Fluorodeoxyglucose (FdG) to image and quantify glucose uptake, which has been found to be correlated with poor prognosis and to be highly dependent on the glycolytic rate [17,35,36].

The PPP, found to be elevated in cancer [37], is a major source of the reduced form of nicotinamide adenine dinucleotide phosphate (NADPH), which is an electron carrier or antioxidant. The PPP involves an oxidative (ox-PPP) and non-oxidative (non-ox-PPP) phase. The non-ox-PPP provides a link to glycolysis as it converts ribose-5-phosphate (R5P) into the glycolytic intermediates fructose-6-phosphate and glyceraldehyde-3-phosphate to promote cellular energy metabolism. This pathway is reversible, being able to redirect the glycolysis intermediates to produce R5P to increase biosynthesis of ribonucleotides when required for proliferation (Figure 3).

A general hypothesis when analyzing glucose metabolism in tumor tissue is that decreasing levels of glucose reflects an increasing energy demand while the degree of lactate production might indicate whether the glucose is guided towards TCA cycle or used for aerobic glycolysis. The decrease in glucose has also been observed by proton HR MR-obtained spectra using tissue extracts from breast cancer [38]. In accordance with a higher energy demand and thereby higher glucose demand in tumors with actively proliferating cells, a previous study reported glucose levels in tissue to be negatively correlated to Ki-67 expression [39]. Among gene expression subtypes, luminal-like xenograft tumors express a higher glycolytic rate compared to more aggressive and fast growing tumors of basal-like xenografts [40]. Further investigation of luminal A tumors from patients suggested glucose concentration as one of the markers for further metabolic subgrouping, where one of the suggested subgroups showed significantly lower concentration of glucose compared to the others [41]. Glucose metabolism does not necessarily behave similarly in all breast cancer gene expression subtypes, potentially contributing to the heterogeneity of the disease. In a more recent study, the main metabolic differences in tumors from untreated breast cancer patients were examined [42]. This revealed three metabolic clusters, where one of the clusters (Mc2) was characterized by high levels of glucose indicative of lower glycolytic rate. In one of the other clusters (Mc3), a higher Warburg effect was evident based on the characteristic low levels of glucose and higher levels of lactate. The gene expression subtypes were evenly distributed among these clusters, suggesting that metabolic differences may provide an additional component of the heterogeneity of breast cancer beyond gene expression.

Although metabolic prediction of treatment response prior to onset of neoadjuvant treatment has currently not been achieved [43,44,45], increase in glucose levels during treatment for responders [44] and 5-year survivors [43] has been observed. If glucose is metabolized though aerobic glycolysis, regardless of oxygen availability, lactate will be produced via the action of LDH. Lactate has been suggested as a key player for cancer development and metastasis [34,46], and HR MAS MR spectroscopy analysis showed lower levels of lactate in ER- breast tumors compared to ER+ tumors [47]. This was later confirmed by MS analysis of breast cancer tissue extracts [48]. In addition, high levels of lactate, together with high glycine levels, has been associated with poor-prognosis for patients with ER+ invasive ductal carcinoma [49]. Accumulation of lactate in tissue extracts analyzed by MR spectroscopy has also been shown to correlate with metastasis [50,51]. Furthermore, in patients diagnosed with locally advanced breast cancer, higher lactate levels prior to treatment start was observed for those who did not survive (5 years), supporting it as a poor-prognosis marker [43,45].

The characteristic of high glycolytic activity is being tested as a target in cancer therapy using different approaches [52]. Direct inhibition of glucose metabolism using the glucose analogue 2-deoxy-D-glucose (2DG) has been extensively studied in cancer cells, especially in combination with other treatments [53,54]. Although preclinical toxicity issues have been a concern regarding this drug, it has been reported as well tolerated in patients [55]. By binding to the glucose transporters, 2DG inhibits glucose uptake and thereby all downstream pathways that rely on glucose to contribute with intermediates in both glycolysis and mitochondrial oxidative phosphorylation. Other possible glycolytic targets include the hexokinases (HKs) where the use of 3-bromopyruvate (3-BrPA) has been found to induce autophagy in breast cancer cell lines [56,57]. Notably, inhibiting glucose metabolism is expected to drive cancer cells to use other sources, e.g., glutamine, for energy and substrate production as a compensatory mechanism. Targeting multiple metabolic pathways simultaneously has therefore been considered an alternative treatment strategy [52].

## 3. Amino Acid Metabolism

Although more than 500 different amino acids exist, only 20 commonly serve as building blocks for proteins in the human body [58]. Amino acids can act as regulators or intermediate metabolites for several important metabolic pathways necessary for cellular maintenance and growth. Cancer cells have been shown to have increased consumption of amino acids and upregulation of corresponding transporters, in addition to altered levels of enzymes that catalyze amino acid synthesis and/or catabolism, thus amino acid metabolism presents several potential targets for cancer treatment and patient stratification [59].

Although glucose is considered the main energy source in human cells, amino acids such as glutamine can be utilized to produce ATP through refilling of intermediates to the TCA cycle. Glutamine is normally considered a non-essential amino acid, however studies have shown that in rapidly dividing cells, including both normal and cancer cells, it is conditionally essential [60]. Glutamine can be transported into the cell, where it is hydrolyzed to glutamate and ammonium by the enzyme glutaminase (GLS or GLS2). Glutamate has several metabolic fates in human cells: protein synthesis, conversion into α-ketoglutarate to enter the TCA cycle, to act as a precursor for the important antioxidant glutathione or to provide the amino group for non-essential amino acids such as alanine, aspartate, serine and glycine [59,60]. Significant amounts of glutamine carbon have been found to be converted to lactate and secreted from cancer cells in vitro, in a similar manner as glucose [17,61]. This reaction involves the malic enzyme and leads to synthesis of CO_2_, pyruvate and NADPH, the latter being a reducing equivalent and as such is important for lipid and nucleotide synthesis. Through this pathway, increased glutaminolysis in proliferative cells can fulfill an important proportion of their NADPH demands [17,61]. An overview of glutamine metabolism is shown in Figure 3.

Increased glutamine transporter activity and GLS expression have been found in several cancers [18,61]. In breast cancer, elevated GLS expression has been associated with high grade and metastatic disease [62,63]. Triple negative breast cancers (TNBC) have been shown to have elevated GLS levels both in vivo and in vitro [63]. In addition, in a study comparing the metabolic profiles of TNBC tissue to triple positive breast cancer, TNBC were shown to have lower glutamine and higher glutamate levels, supportive of increased glutamine metabolism in this subgroup of patients [64]. GLS inhibitors as antitumor treatment for TNBC have shown promising results in preclinical studies, and are currently being tested in clinical trials [63,65].

Glutathione, a tripeptide of glutamate, cysteine and glycine, is a major cellular antioxidant [66], and as such provides protection from reactive oxygen species (ROS) that oxidize and damage cellular proteins, lipids and nucleic acids and may ultimately cause cellular dysfunction or death [67]. It has been hypothesized that high levels of glutathione could contribute to treatment resistance by reducing the effectiveness of drugs intended to damage cancer cells [68]. Additionally, cancer cells with lower levels of glutathione were found to be more sensitive to radiation therapy [68]. Furthermore, in a chemoresistant breast cancer cell line, decreased glutathione levels were suggested to be an essential event in treatment-induced reduction of their resistant properties [69]. In a study investigating the effect of anti-angiogenesis treatment (bevacizumab) of breast cancer in a neoadjuvant setting, lower glutathione levels were detected in patients receiving bevacizumab compared to patients receiving chemotherapy only, suggesting that anti-angiogenesis treatment may induce oxidative stress to promote apoptosis [44].

Taurine is another amino acid whose relevance in cancer has been investigated. However, findings for this metabolite vary with different types of cancer [23], and the involved mechanisms are still unclear. Taurine is not incorporated into proteins, but is still essential with functions related to cell membrane stability and facilitation of ion transport. Elevated levels of taurine in breast tissue compared to normal tissue have been reported in studies using HR MAS MR spectroscopy [70,71]. Taurine has also been negatively associated with axillary lymph node spread [39], and was found to be lower in ER- compared to ER+ tumors [47] and in HER2-positive (HER2+) compared to negative tumors [72]. ER- and HER2+ tumors are generally more aggressive, and when comparing patients with a poor or good prognosis based on clinical parameters (lymphatic spread, tumor size and hormone receptor status) lower taurine levels in tissue from the poor-prognosis patients were detected [39]. A study using two different human breast cancer cell lines showed that treating cells with taurine inhibited growth and induced apoptosis by regulating apoptosis-related proteins of mitochondria [73].

Taurine levels have also shown potential for monitoring treatment response, where breast cancer patients with a clinical response to neoadjuvant doxorubicin treatment had a larger decrease of taurine compared to non-responders [45]. A similar study of breast cancer patients receiving neoadjuvant combinational chemotherapy (FEC-100 and taxanes) with or without bevacizumab also showed decreased taurine levels in responders compared to non-responders after treatment [44]. The finding was however not confirmed in patients receiving epirubiucin and/or paclitaxel, where metabolic profiles could not separate patients with a stable disease from partial responders, possibly due to less extreme response groups in this study [43].

Glycine is a small, non-essential amino acid with important metabolic functions in the human body. Glycine can be derived from glycolysis through its precursor serine from 3-phosphoglycerate, or from choline by oxidation of choline to betaine which is further converted to sarcosine and finally glycine. Mitochondrial serine hydroxymethyltransferase 2 (SHMT2), the primary catalyst for converting serine to glycine, is shown to be one of the most frequently over-expressed metabolic enzymes in human tumors [74].

Glycine has been found to be tumor size-dependent, where tumors larger than 2 cm had significantly higher concentrations of glycine as well as choline compared to smaller tumors [70]. Additionally, glycine has been suggested as a prognostic biomarker in studies comparing tissue samples from patients with a poor or good prognosis based on clinical parameters (lymphatic spread, tumor size and hormone receptor status) [39,72], where high glycine levels were associated with a poor prognosis. Higher levels of glycine were also confirmed in tissue samples from the poor-prognosis basal-like compared to the good-prognosis luminal-like breast cancer xenograft models [75], with gene-expression data suggesting that increased glycine levels were derived from the choline pathway. The hypothesis of glycine levels being predictive for poor prognosis was supported in a study comparing 5-year survivors to non-survivors [49], where non-survivors had higher levels of glycine and lactate in surgically removed tumor tissue. Studies have also shown that non-survivors have higher glycine levels after neoadjuvant treatment compared to surviving breast cancer patients [43,45]. Together, these results suggest high glycine levels in breast cancer to be a marker of poor prognosis.

## 4. Choline Phospholipid Metabolism

**Figure 4:**
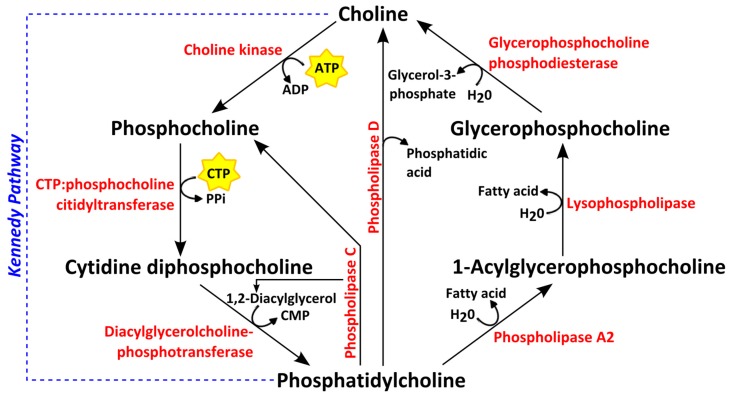
Choline phospholipid metabolism. Enzymes are shown in red. ADP: adenosine diphosphate; ATP: adenosine triphosphate; CMP: cytidine monophosphate; CTP: cytidine triphosphate; PPi: inorganic pyrophosphate.

Choline is an essential organic compound functioning as a precursor for phosphatidylcholine (PtdCho), one of the most abundant phospholipids in eukaryotic cellular membranes [76]. PtdCho is formed de novo from choline via the Kennedy pathway shown in Figure 4. Choline is first transported into the cell and phosphorylated to phosphocholine (PCho) by the enzyme choline kinase. A cytidyldiphosphate (CDP) group is then added to PCho, forming the high-energy donor CDP-choline. To synthetize PtdCho, a lipid anchor such as diacylglycerol (DAG) is added by the enzyme DAG-cholinephosphotransferase [76]. The breakdown products of PtdCho are 1-acylglycerophosphocholine and glycerophosphocholine (GPC), the latter of which is subsequently converted to choline, thereby completing the choline cycle (Figure 4). Since lipid second messengers are synthesized via choline phospholipid metabolism, this pathway plays an additional role in lipid-based signal transduction. Proton MR spectral peaks for Cho, PCho and GPC are detected in vivo as a single peak, i.e., tCho; using ex vivo proton HR MAS MR spectroscopy, however, these choline-containing metabolites can be detected separately and have been found to be expressed at higher levels in cancer than in normal or adjacent non-involved breast tissue [39,70,77].

Choline-containing compounds are considered essential to sustain cell proliferation in terms of being substrates for membrane multiplication for new cells [78,79]. Tumor cells grow rapidly and therefore require high production of phospholipids like PtdCho. The abnormal high production of PtdCho from choline and choline-containing compounds has therefore been studied as an element of cancer metabolic reprogramming for several decades [23] and is an emerging metabolic hallmark for tumor progression [80].

Choline containing metabolite levels have been found to correlate with enzymes involved in choline phospholipid metabolism. Increased tCho and PCho in cancer has been attributed to a higher choline uptake [81,82], as well as increased expression and activity of the enzymes choline kinase alpha (CK-α) [82,83], the isoform which is primarily upregulated in cancers [84] together with phospholipases C [82,85] and D [82,86] and glycerophosphocholine phosphodiesterases [87,88] (Figure 4). The expression of CK-α is shown to correlate with GPC and PCho levels in vitro [85] and ex vivo [89]. The glycerophosphodiester phosphodiesterase domain containing 5 (GDPD5), which encodes for the enzyme glycerophosphocholine phosphodiesterase that catalyzes the degradation of GPC to free choline (Figure 4) has been found to be positively correlated with PCho, tCho and PCho/GPC levels in human breast tumors [87].

Choline phospholipid metabolism has been investigated as a target for antitumor treatment, with CK-α inhibition showing antiproliferative effects in both cancer cell lines and xenograft models of human tumors [90]. Promising results combined with low toxicity profiles for the CK-α inhibitor RSM-932A have led to the drug being currently tested in a phase I clinical trial [91].

Nevertheless, many of the mechanisms governing altered choline phospholipid metabolism in cancer are still not fully understood. Malignant transformation, rather than rapid proliferation, has been suggested as the main driver for abnormal choline metabolism, as non-cancer proliferating cells were found to sustain low PCho, GPC and tCho levels in cell culture [92]. In the same study, the ratio between GPC and PCho was inverted from higher GPC/PCho to higher PCho/GPC with immortalization of cells, suggesting the latter ratio to be a marker for aggressiveness. Similar results were observed in a study comparing triple negative and triple positive cell lines by HR MAS MR spectroscopy of intact cells, where the triple negative cell line had higher PCho levels [93]. In contradiction, ex vivo studies have found GPC/PCho to be elevated in breast cancer subtypes with worse prognosis [47,75]. The discrepancy may be due to the association of higher PCho/GPC to aggressiveness being based on in vitro studies that do not take the effects of the tumor microenvironment into account. Acidic extracellular pH, for example, has been found to significantly increase GPC and decrease PCho levels [94]. Despite of lacking consensus regarding choline metabolite ratios, increased levels of one or more of the individual metabolites constituting tCho are consistently observed in tumors and are associated with cancer aggressiveness. Decreased tCho signal detected in vivo has therefore been suggested as a marker of tumor response to treatment [95,96].

Changes in choline-containing compounds during neoadjuvant chemotherapy have also been detected ex vivo. Cao et al. [43] reported a decreasing trend in GPC levels during treatment, while Euceda et al. [44] detected decreased levels of all the constituents of tCho. In the latter study, choline and PCho were also significantly lower in patients exhibiting a good pathological response compared to pathological non-responders at the end of treatment. These findings point to a decrease in choline phospholipid metabolism as an effect of breast cancer therapy, which appears more prominent in responders. This decrease may reflect an attenuation of malignancy and aggressiveness in the treated tumors. Metabolic profiles of intact breast tumor tissue have provided indications that changes in choline phospholipid metabolism during treatment are associated with prognosis. In patients diagnosed with locally advanced breast cancer receiving neoadjuvant chemotherapy [45], long-term survivors (≥5 years) had higher levels of tCho pre-treatment when compared to non-survivors. In a larger study [43], long-term survivors, but not non-survivors, exhibited a significant decrease in GPC as an effect of neoadjuvant chemotherapy. Elucidating the molecular basis underlying the tCho changes in tumors responding to treatment may therefore be useful in improving patient outcome.

Choline phospholipid metabolism seems to be a factor contributing to breast cancer heterogeneity determined at different molecular levels. Regarding the protein level, ER+ tumors have been associated with higher PCho and lower GPC and choline levels compared to ER- tumors [47]. In the previously mentioned study defining three metabolic clusters of breast cancer [42], one of the clusters (Mc1) was defined by significantly higher levels of GPC and PCho and exhibited higher levels of the gene for CK-α. Integrated pathway analysis identified glycerophospholipid metabolism as the most significantly different pathway between this cluster and the others.

Distinct differences in choline metabolite profiles have been observed between luminal-like and basal-like xenografts. Moestue et al. [75] found that the more aggressive basal-like tumor xenografts had a higher GPC and lower PCho concentration than the less aggressive luminal-like xenograft models. To evaluate how well the models represent human breast cancer, GPC and PCho levels in breast cancer tissue from patients were examined. A higher GPC/PCho ratio in triple negative compared to ER+/PgR+ breast cancer was observed, in concordance to the basal-like and luminal-like subtypes, respectively. The same GPC and PCho trends between the gene expression subtypes was observed in two other separate studies [40,89].

Differences in how these choline-containing compounds are affected by treatment have also been observed between basal-like and luminal-like xenografts. Moestue et al. [97] found that treatment with inhibitors of the PI3K pathway significantly increased PCho in basal-like, but not in luminal-like, tumor xenografts, which coincided with decreased proliferation. This suggests that breast cancer heterogeneity at the metabolic level could be exploited to further stratify treatment and to determine new possible drug targets for specific patient subgroups. When looking into a heterogeneous panel of TNBC xenograft models, however, Euceda et al. [98] found that PCho levels were significantly lower in xenografts treated with an mTOR/AKT/PI3K inhibitor than in untreated controls. This can be considered contradictory to the increased PCho levels in basal-like xenografts after treatment with PI3K inhibitors in [97], since there is a high degree of overlap between the basal-like and TNBC subtypes. In general, tCho response to breast cancer treatment seems to depend not only on subtype, but also on factors such as the drug being administered and the time period following treatment when the metabolites are measured. However, the distinct differences in choline metabolite profiles observed in luminal-like and basal-like xenografts have also been found to correlate with gene expression differences [75,89], indicating the need to combine data levels for better breast cancer stratification.

## 5. Conclusions

Metabolic profiles of breast tumors can be generated from intact tissue using proton HR MAS MR spectroscopy. This technique has been applied to study metabolites involved in the cancer-relevant pathways of glucose metabolism, amino acid metabolism and choline phospholipid metabolism. This review has provided a description of the said pathways as well as an overview of the main metabolic findings within them concerning breast cancer, focusing on proton MR detectable metabolites in intact tumor tissue, supported by in vitro and MS findings. HR MAS MR spectroscopy has proved to be a valuable tool in the characterization of breast cancer, with an important benefit being its translational potential to the in vivo setting. Since metabolites may serve as phenotypic markers resulting from both genome and proteome alterations, MR metabolomics can potentially be used to provide important predictive and prognostic information. Future studies combining metabolic profiles with data from other platforms could potentially lead to a more refined stratification of breast cancer patients and improved treatment strategies targeting metabolic pathways.
